# Supplementary material for: Control of multidimensional systems on complex network
Source: PLoS One. 2017 Sep 11;12(9):e0184431. doi: 10.1371/journal.pone.0184431 (PMC5593194; doi:10.1371/journal.pone.0184431)
Supplement: S1 File — (PDF) [file pone.0184431.s001.pdf]

Control of multidimensional systems on complex network: Supplementary Information

Giulia Cencetti,[1,2,3] Franco Bagnoli,[2,3] Giorgio Battistelli,[1] Luigi Chisci,[1] and Duccio Fanelli[2,3]

[1] *Dipartimento di Ingegneria dell'Informazione, Universita' di Firenze, Via S. Marta 3, 50139 Florence, Italy*

[2] *Dipartimento di Fisica e Astronomia and CSDC, Università degli Studi di Firenze, via G. Sansone 1, 50019 Sesto Fiorentino, Italia*

[3] *INFN Sezione di Firenze, via G. Sansone 1, 50019 Sesto Fiorentino, Italia*

I. GENETIC NETWORK MODEL

We shall here justify the model of genetic regulatory network analyzed in the main text. Consider first a small regulatory network consisting of one gene (whose activity is labelled x) and one protein (associated to the continuous concentration y). A positive regulation loop can be modeled as:

$$\begin{aligned} \dot{x} &= k_1 g(y) - \gamma_1 x \\ \dot{y} &= k_2 x - \gamma_2 y \end{aligned}$$

where:

$$g(y) = \frac{y^n}{K + y^n}. \tag{S1}$$

In the following, we will make the choice $n = 2$ and $K = 1$. Similarly, a negative regulation loop can be modeled as:

$$\begin{aligned} \dot{x} &= k_1 (1 - g(y)) - \gamma_1 x \\ \dot{y} &= k_2 x - \gamma_2 y \end{aligned}$$

As it should be, the concentration of proteins grows with the level of gene activity. As a first step approximation, and to avoid dealing with two distinct families of mutually interlinked constituents (genes and proteins), we can replace y with x in the argument of $g(\cdot)$, via adiabatic elimination (apart from redefinition of the involved constants). Building on the above we model the extended regulatory network as:

$$\dot{x}_i = k_i \sum_j A_{ij} g(x_j) - \gamma_i x_i + k_i \eta_i \tag{S2}$$

where x_i stands for the activity of gene i. For *positive* feedbacks between species i and j, $A_{ij} = 1$, while, for *negative* loops $A_{ij} = -1$. The parameter η_i stands for the number of negative loops (number of negative entries of the i-th row of $\boldsymbol{A}$) that are associated to node i. Finally, to keep the structure as simple as possible we set $k_i = 1$ for all i. The matrix $\boldsymbol{A}$ employed in the example reported in the main body has a simple structure and it has been chosen for purely illustrative purposes: it represents a regular tree network with branching ratio $r = 4$.

II. CONTROLLING THE MICROBIOTA NETWORK: THE EXPERIMENTAL DATA

The second application of the proposed control method deals with a model of gut microbiota. As explained in the main body of the paper, the model is well established and builds on a generalization of the celebrated Lotka-Volterra equations:

$$\dot{x}_i = x_i(r_i - s_i x_i) + x_i \sum_{j \neq i} A_{ij} x_j \qquad i = 1, \ldots, N \tag{S3}$$

The above equations take into account species-species interactions and self-regulation, this latter effect being described in terms of a logistic growth. Despite its intrinsic simplicity, the model is often invoked to explain how ecological

interactions, e.g., mutualism and competition for nutrients, can lead to complex phenomena, as multi-stability or antibiotic mediated catastrophic losses of biodiversity. In [?] the model has been applied to a relatively small (mice gut) microbiota system made up of 11 distinct populations. More precisely, the ten most abundant species have been identified: all together they account for the vast majority ($\sim 90\%$) of the total populations found in the mice gut. The remaining populations are grouped into a unique (non-homogeneous) category referred to as “Other”. The authors of [?] analyzed the experiments reported in [?] and provided a quantitative characterization of the coefficients that enter the definition of the relevant quantities $\boldsymbol{r}$, $\boldsymbol{s}$ and $\boldsymbol{A}$. These latter are reported in table (S4) together with the names of the involved species:

Populations	$\boldsymbol{r}$	$\boldsymbol{s}$	$\boldsymbol{A}$										
Barnesiella	0.368	0.205	0	0.0984	0.167	-0.165	-0.143	0.0199	-0.515	-0.392	0.346	0.00888	-0.269
undefined genus of Lachnospiraceae	0.310	0.105	0.0621	0	-0.0430	-0.155	-0.187	0.0270	-0.459	-0.414	0.301	0.0221	-0.196
unclassified Lachnospiraceae	0.356	0.102	0.144	-0.192	0	-0.140	-0.165	0.0136	-0.504	-0.772	0.292	-0.00596	-0.206
Other	0.540	0.831	0.224	0.138	0.000459	0	-0.224	0.220	-0.205	-1.01	0.666	-0.0390	-0.400
Blautia	0.709	0.709	-0.180	-0.0513	$-5{,}03\times10^{-5}$	-0.0542	0	0.0162	-0.507	0.554	0.157	0.224	0.10635
undefined genus of unclassified Mollicutes	0.471	0.423	-0.111	-0.037	-0.0426	0.0410	0.261	0	-0.185	-0.4326	0.165	-0.0610	-0.265
Akkermansia	0.230	1.21	-0.127	-0.186	-0.122	0.381	0.400	-0.161	0	1.390	-0.379	0.192	-0.0963
Coprobacillus	0.830	4.35	-0.0712	6.04×10^{-4}	0.0803	-0.455	-0.503	0.169	-0.562	0	0.443	-0.223	-0.207
Clostridium difficile	0.392	0.0558	-0.0375	-0,0333	-0.0499	-0.0904	-0.102	0.0323	-0.182	-0.303	0	0.0144	-0.00767
Enterococcus	0.291	0.192	-0.0422	-0.0131	0.0240	-0.118	-0.329	0.0207	0.0548	-2.10	0.111	0	0.0238
undefined genus of Enterobacteriaceae	0.324	0.384	-0.374	0.278	0.249	-0.168	0.0840	0.0337	-0.232	-0.395	0.314	-0.0388	0

(S4)

The concentration of the species are measured in 10^{11} rRNAcopies/cm^3, the coefficients $\boldsymbol{r}$ have the dimension of the inverse of time (measured in days), and $\boldsymbol{s}$ is expressed as the inverse of the product of a time for a concentration.

The second application discussed in the main body of the paper, i.e. that targeted to controlling the microbiota dynamics, assumes the above parameters. Notice that the set of considered species includes the spore-forming pathogen *Clostridium difficile*. To lower its concentration (and so diminish the probability of infection) is one of the goals of the implemented control. As explained in the paper, three different control schemes are considered. In the following we will provide some additional information for each of the analyzed schemes. The physical dimension of the inserted controller u is again 10^{11} rRNAcopies/cm^3. The parameters $\boldsymbol{\beta}$ have dimension of the inverse of a time, while ρ is a-dimensional.

A. Case A: Stabilizing an unstable fixed point by means of an external controller

The first of the three different control strategies for the microbiota network discussed in the main body involves only 5 out of the 11 populations being examined (more precisely *Barnesiella*, *Blautia*, und. Mollicutes, *Coprobacillus* and und. Enterobacteriaceae). An unstable fixed point of equation (S3), which contains only these latter species, has been calculated and denoted by $\bar{\boldsymbol{x}}$ (see table (S5)). The maximum eigenvalue of the Jacobian matrix evaluated at $\bar{\boldsymbol{x}}$ is $(\lambda_{Re})_{max} = 0.0148 > 0$, thus implying instability. As discussed in the main body of the paper, it is possible to stabilize a different equilibrium, denoted by $\boldsymbol{x}^*$ in (S5), which corresponds to a slight modification of the unstable solution $\bar{\boldsymbol{x}}$. Following the procedure detailed in the paper one can readily calculate the parameters $\boldsymbol{\alpha}$ and $\boldsymbol{\beta}$ (S5). The values obtained are reported in table (S5). Direct simulations of the controlled system, as displayed in Figure S1, confirms that stability has been indeed achieved.

Populations	$\bar{\boldsymbol{x}}$	$\boldsymbol{x}^*$	$\boldsymbol{\alpha}$	$\boldsymbol{\beta}\ \times10^4$
Barnesiella	0.9736	0.9917	0.0186	0.1860
Blautia	0.8840	0.9093	0.0089	-0.0638
und. uncl. Mollicutes	1.2361	1.2396	0.0089	-0.0032
Coprobacillus	0.1169	0.1363	0.1005	0.0495
und. Enterobacteriaceae	0.0756	0.0894	0.0175	-2.2067

(S5)

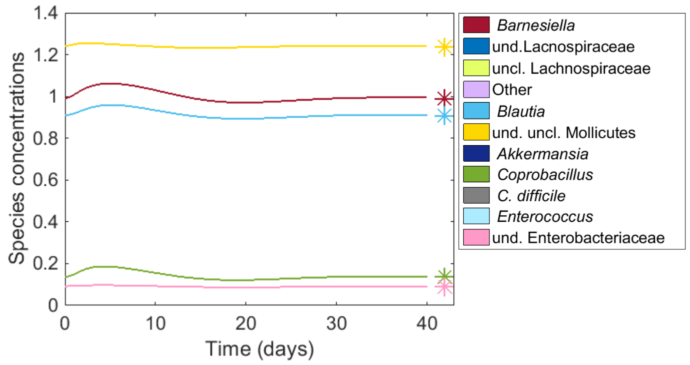

Figure S1: Numerical integration of the controlled system (see equations (2) in the main body). The equilibrium state stabilized upon injection of the controller (stars) is a slight modified version of the initially unstable fixed point, see table (S5). The system is initialized out of equilibrium and, after a transient, converges to $\boldsymbol{x}^*$.

B. Case B: Acting with one species of the pool to damp the concentration of the pathogens

Consider the stable fixed point $\boldsymbol{x}^*$ (table (S6)) composed by *Barnesiella*, und. Lachnospiraceae, Other, *Blautia* and *C. difficile*. As explained in the paper, we now insert in the system another population, selected from the extended pool of interacting species. This latter configures as the controller. Vector $\boldsymbol{\alpha}$ therefore follows from the interaction matrix $\boldsymbol{A}$. One can then calculate the fixed point that can be eventually attained by the system, given the specific selected controller. Retaining only the meaningful cases (fixed points with all positive entries), we obtain three possible solutions: $\boldsymbol{x}^*_L$ where the added species is uncl. Lachnospiraceae, $\boldsymbol{x}^*_M$ adding as external control the und. uncl. Mollicutes and $\boldsymbol{x}^*_E$ adding und. Enterobacteriaceae (see table S6). From inspection of the obtained solutions, one can appreciate the impact of the different controllers employed: in the latter case the concentration of *C. difficile* stays almost constant, in the second example it increases, while in the first case it is reduced by a significant amount.

Populations	$\boldsymbol{x}^*$	$\boldsymbol{x}^*_L$	$\boldsymbol{x}^*_M$	$\boldsymbol{x}^*_E$
Barnesiella	2.0745	2.5166	2.0480	2.2542
und. Lachnospiraceae	2.3607	1.9876	2.2674	2.7007
Other	1.9608	1.6929	1.9252	2.0842
Blautia	0.2724	0.1422	0.6171	0.1345
C. difficile	0.5402	0.2435	0.6194	0.5259

(S6)

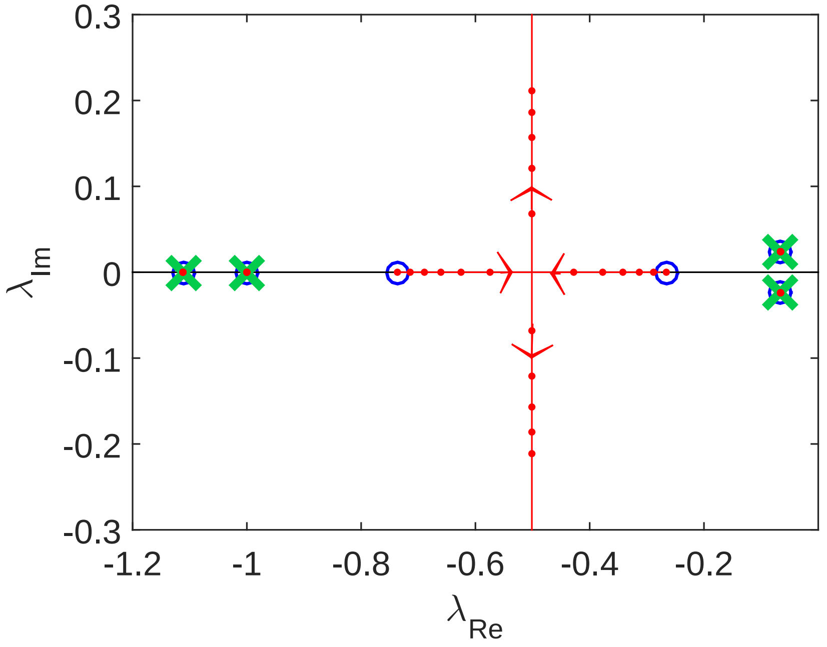

Figure S2: Root locus diagram relative to the Case B where, starting from the pool of 5 populations the control is performed adding the species of uncl. Lachnospiraceae. Blue circles correspond to the position in the complex plane of the roots of $\mathcal{D}(\lambda)$ (eigenvalues of $\boldsymbol{J}$ when $\rho = 0$) while green crosses indicate the eigenvalues of the Jacobian in the limit $\rho \to \infty$. Paths followed by the eigenvalues when progressively increasing ρ are shown by the red lines, while the red dots represent the solutions for discrete values of ρ, scanning the interval from 0 to 0.1.

C. Case C: Driving to extinction one species, the other being the target of the control

In this case we aim at enforcing the extinction of one of the species (here *C. difficile*), acting on the other ones. In other words the parameter vector $\boldsymbol{\alpha}$ (which characterizes the action of the control against the species) is forced to have the component relative to *C. difficile* equal to zero. At the same time, the corresponding entry of the vector $\boldsymbol{x}^*$ to be eventually stabilized is set to a negligible value. The concentrations of the species for respectively the initial and the final fixed points are compared in Figure 2 of the main body. The corresponding values of $\boldsymbol{\alpha}$ are also plotted. As a supplementary material, we here report in Figure S3 the root locus diagram obtained for the case at hand.

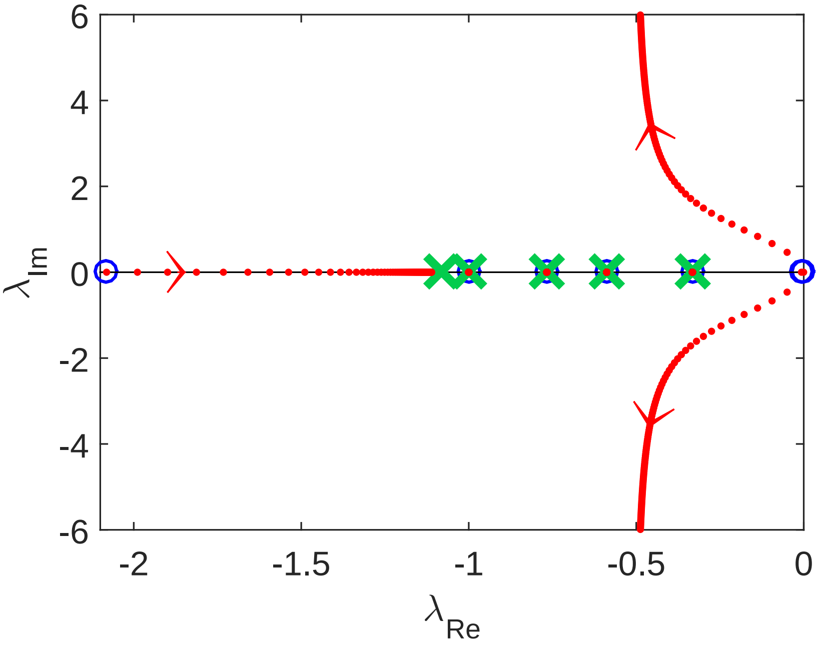

Figure S3: Root locus diagram relative to the Case C, where an indirect control is applied on the reduced system of 6 species. For a description of the symbols, refer to the caption of Figure S2.

III. EXPLOITING A TRANSIENT CONTROL TO DRIVE THE SYSTEM TOWARDS AN EXISTING STABLE FIXED POINT

As anticipated in the main body of the paper, the control can be effectively employed to steer the system towards a stable fixed point of the unperturbed dynamics, starting from out-of-equilibrium initial conditions. In this case, we set $\dot{u} = -\gamma u - \sum_j \beta_j (x_j - x_j^*)$. Here, x_j^* is an equilibrium solution of the uncontrolled dynamics, which proves linearly stable to external perturbations. The parameter γ can be tuned as desired so as to help the convergence towards x_j^* without falling in the basin of attraction of other existing fixed points. As a proof of principle of the method, we choose a stable fixed point of the global macrobiota ecosystem, i.e. including the complete pool of 11 populations. This is characterized by $x_1^* = 9.299$, $x_3^* = 12.3085$, $x_4^* = 3.1627$ and $x_j^* = 0$ for $j \neq 1, 3, 4$. The largest real part of the eigenvalues of the associated 11×11 Jacobian matrix turns out to be $\langle \lambda_{Re} \rangle = -0.1306 < 0$, thus implying stability of the aforementioned equilibrium. Imagine to initialize the system out of equilibrium with all species, including the pathogen *C. difficile*, being assigned a random concentration $x_j(0) \neq 0$. The system is let evolve for a while and then, at time t^*, the control is injected. Here, $\boldsymbol{\alpha}$ and $\boldsymbol{\beta}$ are assigned as random, uniformly distributed over [0,1], parameters. As clearly displayed in Figure S4, the system is steadily moved towards the equilibrium x_j^* (stars), while the control converges to zero after an abrupt jump. In other words, after a transient whose duration depends on the chosen parameters, the system achieves its asymptotic (pathogen free) equilibrium and the control can be safely disconnected.

IV. ON THE CONTROLLABILITY CONDITION

Observe that the control procedure here discussed requires computing the vector $\boldsymbol{\beta}$. Determining this latter implies inverting a matrix, an operation that imposes a mathematical constraint that we shall hereafter analyze more in depth. The matrix to be inverted, as defined in the main text, reads:

$$H_{nm} = -\sum_{k=0}^{N-n} c_{k+n+1} (\boldsymbol{G}^k \boldsymbol{q})_m \tag{S7}$$

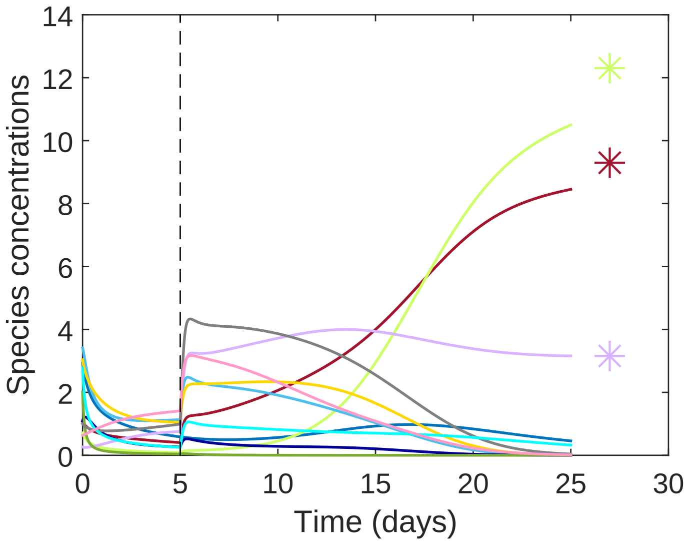

(a)

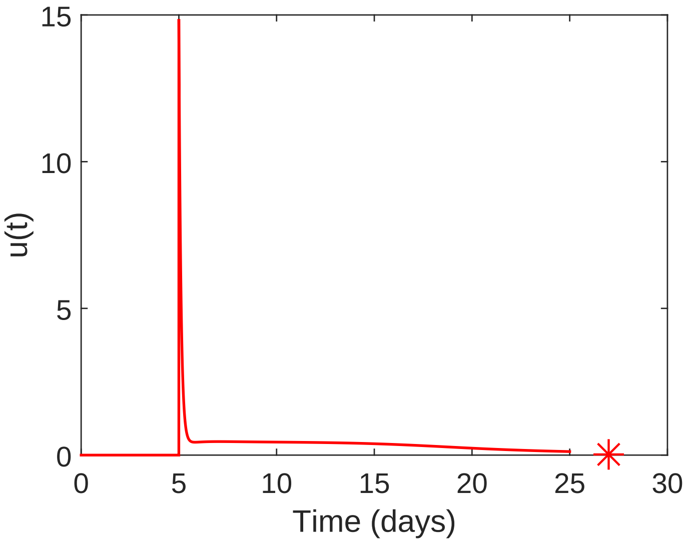

(b)

Figure S4: Driving the system towards a stable fixed point of the unperturbed dynamics. The system is initiated out-of-equilibrium: the concentration of all species, including the pathogen *C. difficile*, is set to values different from zero. At $t^* = 5$ days the control u is injected. After a transient the system converges to its stable equilibrium characterized by $x_1^* = 9.299$, $x_3^* = 12.3085$, $x_4^* = 3.1627$ and $x_j^* = 0$ for $j \neq 1, 3, 4$, while the control u is turned to zero. Here $\gamma = 10$ and $u(0) = 15$.

where c stands for the coefficients[1] of the characteristic polynomial of $\boldsymbol{G}$, namely $\det(\boldsymbol{G}-\lambda\boldsymbol{I})=\sum_{l=0}^{N}c_{l+1}\lambda^l$. The invertibility is ensured if $\det(\boldsymbol{H})\neq 0$, in formulae:

$$\begin{aligned}\det(\boldsymbol{H}) &= \sum_{i_1=1}^{N}\cdots\sum_{i_N=1}^{N}\epsilon_{i_1,\dots i_N}H_{1i_1}H_{2i_2}\dots H_{N-1i_{N-1}}H_{Ni_N} = \\ &= \sum_{i_1=1}^{N}\cdots\sum_{i_N=1}^{N}\epsilon_{i_1,\dots i_N}\left[-\sum_{k_1=0}^{N-1}c_{k_1+2}(\boldsymbol{G}^{k_1}\boldsymbol{q})_{i_1}\right]\left[-\sum_{k_2=0}^{N-2}c_{k_2+3}(\boldsymbol{G}^{k_2}\boldsymbol{q})_{i_2}\right]\dots \\ &\dots\left[-\sum_{k_{N-1}=0}^{1}c_{k_{N-1}+N}(\boldsymbol{G}^{k_{N-1}}\boldsymbol{q})_{i_{N-1}}\right][-c_{N+1}q_{i_N}]\neq 0\end{aligned} \tag{S9}$$

where $\epsilon_{i_1,\dots i_N}$ is the Levi-Civita tensor. This complicated expression can be heavily simplified. The Levi-Civita symbol is in fact totally antisymmetric in the permutation of its indices. As a consequence, all the terms multiplied by $\epsilon_{i_1,\dots i_N}$ which are symmetric in the permutations, cancel out. It follows that the terms containing the product of two or more factors $(\boldsymbol{G}^k\boldsymbol{q})$ with the same power k are identically equal to zero. The only terms which surviveare those obtained by just retaining the largest possible value of$k$ in each summation ($k_1=N-1$, $k_2=N-2$,..., $k_{N-1}=1$). In formulae:

$$\begin{aligned}\det(\boldsymbol{H}) &= (-1)^N\sum_{i_1=1}^{N}\cdots\sum_{i_N=1}^{N}\epsilon_{i_1,\dots i_N}c_{N+1}(\boldsymbol{G}^{N-1}\boldsymbol{q})_{i_1}c_{N+1}(\boldsymbol{G}^{N-2}\boldsymbol{q})_{i_2}\dots c_{1+N}(\boldsymbol{G}\boldsymbol{q})_{i_{N-1}}c_{N+1}q_{i_N} = \\ &= (-1)^N(-1)^{N^2}\sum_{i_1=1}^{N}\cdots\sum_{i_N=1}^{N}\epsilon_{i_1,\dots i_N}(\boldsymbol{G}^{N-1}\boldsymbol{q})_{i_1}(\boldsymbol{G}^{N-2}\boldsymbol{q})_{i_2}\dots(\boldsymbol{G}\boldsymbol{q})_{i_{N-1}}q_{i_N}.\end{aligned} \tag{S10}$$

where use has been made of the fact that $c_{N+1}=(-1)^N$ (see equation (S8) in the footnote 1).

Drawing on this preliminary observations it is possible to re-interpret the above controllability constraint, making contact with standard control theory. The controllability condition amounts to require that the matrix

$$\boldsymbol{C}\equiv[\boldsymbol{q},\boldsymbol{G}\boldsymbol{q},...,\boldsymbol{G}^{N-1}\boldsymbol{q}]=\begin{pmatrix} q_1 & (\boldsymbol{G}\boldsymbol{q})_1 & (\boldsymbol{G}^2\boldsymbol{q})_1 & \dots & (\boldsymbol{G}^{N-1}\boldsymbol{q})_1 \\ q_2 & (\boldsymbol{G}\boldsymbol{q})_2 & (\boldsymbol{G}^2\boldsymbol{q})_2 & \dots & (\boldsymbol{G}^{N-1}\boldsymbol{q})_2 \\ \vdots & & & & \\ q_N & (\boldsymbol{G}\boldsymbol{q})_N & (\boldsymbol{G}^2\boldsymbol{q})_N & \dots & (\boldsymbol{G}^{N-1}\boldsymbol{q})_N \end{pmatrix} \tag{S11}$$

has maximum rank. Here, with the notation $(\boldsymbol{G}^l\boldsymbol{q})_k$ we identify the k-th entry of the vector obtained from the product of the matrix $\boldsymbol{G}$ to the power of l with vector $\boldsymbol{q}$. In system theory the matrix $\boldsymbol{C}$ is called the *controllability* matrix of the pair $(\boldsymbol{G},\boldsymbol{q})$. Since $\boldsymbol{C}$ is a square matrix, the maximum rank condition is equivalent to require $\det(\boldsymbol{C})\neq 0$, in

---

[1]

$$\begin{aligned} c_1 &= \det(\boldsymbol{G}) \\ c_k &= \frac{(-1)^{k-1}}{(k-1)!}\sum_{\substack{j_1\cdots j_{k-1}=1 \\ j_1\neq\cdots\neq j_{k-1}}}^{N}\det\boldsymbol{G}_{(j_1j_1)(j_2j_2)\dots(j_{k-1}j_{k-1})}\ \text{for}\ k=2,\dots,N-1 \\ c_N &= (-1)^{N-1}Tr(\boldsymbol{G}) \\ c_{N+1} &= (-1)^N \end{aligned} \tag{S8}$$

where with $\boldsymbol{G}_{(j_1j_1)(j_2j_2)\dots(j_{k-1}j_{k-1})}$ we identify the minor obtained from matrix $\boldsymbol{G}$ by removing the j_1-th, j_2-th,...,j_{k-1}-th rows and columns.

formulae:

$$\det(\boldsymbol{C}) = \sum_{\sigma_1=1}^{N} \cdots \sum_{\sigma_N=1}^{N} \epsilon_{\sigma_1,\ldots,\sigma_N} C_{\sigma_1 1} C_{\sigma_2 2} \ldots C_{\sigma_{N-1} N-1} C_{\sigma_N N} =$$
$$= \sum_{\sigma_1=1}^{N} \cdots \sum_{\sigma_N=1}^{N} \epsilon_{\sigma_1,\ldots,\sigma_N} (\boldsymbol{G}^0 \boldsymbol{q})_{\sigma_1} (\boldsymbol{G}^1 \boldsymbol{q})_{\sigma_2} \ldots (\boldsymbol{G}^{N-2} \boldsymbol{q})_{\sigma_{N-1}} (\boldsymbol{G}^{N-1} \boldsymbol{q})_{\sigma_N} = \tag{S12}$$
$$= (-1)^N \sum_{\sigma_1=1}^{N} \cdots \sum_{\sigma_N=1}^{N} \epsilon_{\sigma_1,\ldots,\sigma_N} (\boldsymbol{G}^{N-1} \boldsymbol{q})_{\sigma_1} (\boldsymbol{G}^{N-2} \boldsymbol{q})_{\sigma_2} \ldots (\boldsymbol{G} \boldsymbol{q})_{\sigma_{N-1}} (\boldsymbol{q})_{\sigma_N} \neq 0$$

where use has been made of the definition of $\boldsymbol{C}$ (S11), namely $C_{ij} = (\boldsymbol{G}^{j-1}\boldsymbol{q})_i$.
Expressions (S10) and (S12) are identical (except for the sign) and consequently the two conditions, $\det(\boldsymbol{H}) \neq 0$ e $\det(\boldsymbol{C}) \neq 0$, prove equivalent. Stated differently, the condition of invertibility of matrix $\boldsymbol{H}$, obtained as a self-consistent constraint for the introduced control scheme, coincides with the standard controllability condition, as known in control theory.
As a final remark we recall (see main body) that our goal is not to arbitrarily assign the polynomial $\mathcal{N}(\lambda)$ but rather to locate its roots z_k within the open left-hand plane. In this respect, the necessary and sufficient system-theoretic condition is the so-called *stabilizability* of the pair $(\boldsymbol{G}, \boldsymbol{q})$, which results in the Popov-Belevitch-Hautus rank condition [**? ?**].
